# The Editor's Letter-Box

**Published:** 1915-02-06

**Authors:** 


### THE EDITOR'S LETTER-BOX.

### The Hampstead Appointment.

*To the Editor of* The Hospital.

Sir,—The Council of the Hampstead General Hospital, when inviting applications for the post of secretary, expressly stated that "Candidates must have had previous hospital experience."

Whilst fully acknowledging that the Council had every right to appoint whom they would, it does not appear to me, in the face of the above clause, that they have "played the game" in electing a man without such experience.—I am, your obedient servant,

Fair Play.

